# Photosynthesis acclimation under severely fluctuating light conditions allows faster growth of diatoms compared with dinoflagellates

**DOI:** 10.1186/s12870-021-02902-0

**Published:** 2021-04-01

**Authors:** Lu Zhou, Songcui Wu, Wenhui Gu, Lijun Wang, Jing Wang, Shan Gao, Guangce Wang

**Affiliations:** 1 CAS and Shandong Province Key Laboratory of Experimental Marine Biology, Center for Ocean Mega-Science, Institute of Oceanology, Chinese Academy of Sciences, Qingdao, 266071 China; 2 Laboratory for Marine Biology and Biotechnology, Qingdao National Laboratory for Marine Science and Technology, Qingdao, 266237 China; 3 College of Earth Sciences, University of Chinese Academy of Sciences, Beijing, 100049 China

**Keywords:** Diatoms, Dinoflagellates, Fluctuating light, Pioneer bloom, PGR5, *Phaeodactylum tricornutum*, Phytoplankton bloom succession

## Abstract

**Background:**

Diatoms contribute 20% of the global primary production and are adaptable in dynamic environments. Diatoms always bloom earlier in the annual phytoplankton succession instead of dinoflagellates. However, how diatoms acclimate to a dynamic environment, especially under changing light conditions, remains unclear.

**Results:**

We compared the growth and photosynthesis under fluctuating light conditions of red tide diatom *Skeletonema costatum*, red tide dinoflagellate *Amphidinium carterae*, *Prorocentrum donghaiense*, *Karenia mikimotoi*, model diatom *Phaeodactylum tricornutum*, *Thalassiosira pseudonana* and model dinoflagellate *Dinophycae Symbiodinium*. Diatoms grew faster and maintained a consistently higher level of photosynthesis. Diatoms were sensitive to the specific inhibitor of Proton Gradient Regulation 5 (PGR5) depending photosynthetic electron flow, which is a crucial mechanism to protect their photosynthetic apparatus under fluctuating light. In contrast, the dinoflagellates were not sensitive to this inhibitor. Therefore, we investigate how PGR5 functions under light fluctuations in the model diatom *P. tricornutum* by knocking down and overexpressing PGR5. Overexpression of PGR5 reduced the photosystem I acceptor side limitation (Y (NA)) and increased growth rate under severely fluctuating light in contrast to the knockdown of *PGR5*.

**Conclusion:**

Diatoms acclimatize to fluctuating light conditions better than dinoflagellates. PGR5 in diatoms can regulate their photosynthetic electron flow and accelerate their growth under severe light fluctuation, supporting fast biomass accumulation under dynamic environments in pioneer blooms.

**Supplementary Information:**

The online version contains supplementary material available at 10.1186/s12870-021-02902-0.

## Background

Blooms during the upwelling season provide productivity in the ocean for a year and promote the global biochemical cycle [1, 2]. In early spring, pioneer diatoms bloom primarily from melting sea ice or sediment that was brought to the euphotic zone [3–5]. With diatom bloom recession, the matter and energy are converted to ocean sediment and subsequent blooms of dinoflagellates or zooplankton [3, 6–8]. However, the mechanism of the annual bloom succession between diatoms and dinoflagellates remains unclear.

Many studies along with analyses of monitoring data have been conducted to determine the imperative factor in the sequential bloom. A key reason for replacement of diatoms with dinoflagellates is nutrition [9–11]. Reduced dissolved silicate accelerates the decline of diatom biomass, and low nutrition increases the competitiveness of dinoflagellates [9–13]. Sufficient silicate is necessary (but not determinate) for the formation of diatom blooms [9, 14]. The temperature in late winter is also insufficient for diatoms to bloom in this season [2, 15, 16]. Some researchers have suggested that light [1, 2, 14–17] and wind [18–20] can be the stimuli for prolific diatoms, which needs further verification. However, these independent factors may not be applicable in all situations [14, 15, 21]. Therefore, to date, it is uncertain why diatom blooms form large-scale biomass in the short term and which factor signals the arrival of the blooms. Light fluctuation as a result of wind, turbulence, upwelling systems, waves, and surface lens effects [22–24] is an important environmental factor that has been monitored but ignored in ocean surveys [14, 15].

Notably, diatoms prefer and adapt to these dynamic environments, especially fluctuating light conditions. A temporarily enhanced wind can accelerate the growth of diatoms within a few days [18, 19]. In addition, diatoms are more adaptable in turbulent environments than dinoflagellates [3, 25, 26]. Reduced turbulence after stratification was considered a precondition of dinoflagellate bloom [25, 27]. Further, greater wave heights in winter and spring imply a more severe light fluctuation for diatoms. Pioneer diatoms are blown into shallow water, and blooms primarily occur within 50 m from the surface and could even concentrate on the surface [4, 12–14, 17, 28–30], whereas dinoflagellate blooms are usually in the subsurface, deeper than 10 m [1, 31–33]. The light intensity at the sea surface reaches 2000 μmol m^− 2^ s^− 1^ or sometimes more [14, 33, 34]. However, the illumination can sometimes be reduced to 1% at a depth of approximately 10 m [14, 15, 31, 33], especially for diatom blooms [14]. Finally, diatoms show absolute dominance in the upwelling system [35–45]. Diatoms can dominate in high-intensity upwelling regions and during upwelling stress periods [45–47]. In contrast, dinoflagellates are rich in stable regions and can only tolerate moderate and peripheral upwelling regions [40], an upwelling relaxation period [40, 45, 48, 49], or transitional period to downwelling [45, 50]. Besides nutrition and temperature, other more complex factors could influence the succession of diatom and dinoflagellate blooms. These special and changeable conditions imply that diatoms face complicated or multiple stresses, especially fluctuating light [14, 15].

Knowledge about the biological mechanism of bloom succession remains limited. Diatom was considered having special, efficient and well-adapted photosynthesis [51–53]. The fucoxanthin-Chl a/c complex (FCP), which was unique in diatom, can efficiently harvest light energy under low light and dissipate excess energy under high light [54]. However only few studies have focused on how these ocean photosynthetic organisms respond to light fluctuation [55]. In the current study, the growth and photosynthesis of three diatoms and four dinoflagellates under mildly fluctuating light (mFL) and severely fluctuating light (sFL) conditions were compared. The diatoms showed higher growth rates and more stable photosynthetic activity than the four dinoflagellates under the fluctuating light. The diatoms were also found to be sensitive to the inhibitor of Proton Gradient Regulation (PGR)5/PGRL1-dependent cyclic electron flow (CEF), a potential pathway responding to light fluctuations [56–58], whereas the dinoflagellates were not in this work.

*Phaeodactylum tricornutum* was used as a model to investigate how diatoms respond to fluctuating light in the ocean due to the availability of its complete genome sequence and pre-developed transformation techniques [59–61]. Overexpression of *PGR5* could accelerate growth and decrease PSI accepted side limitations only under sFL, which was different from the findings of previous studies on photosynthesis in land and fresh water. Our results suggest that special photosynthesis in marine diatoms makes them grow better and acclimate to the turbulent sea surface and strong upwelling system.

## Results

### Growth and photosynthetic activities in marine diatoms and dinoflagellates under fluctuating light

**Fig. 1:**
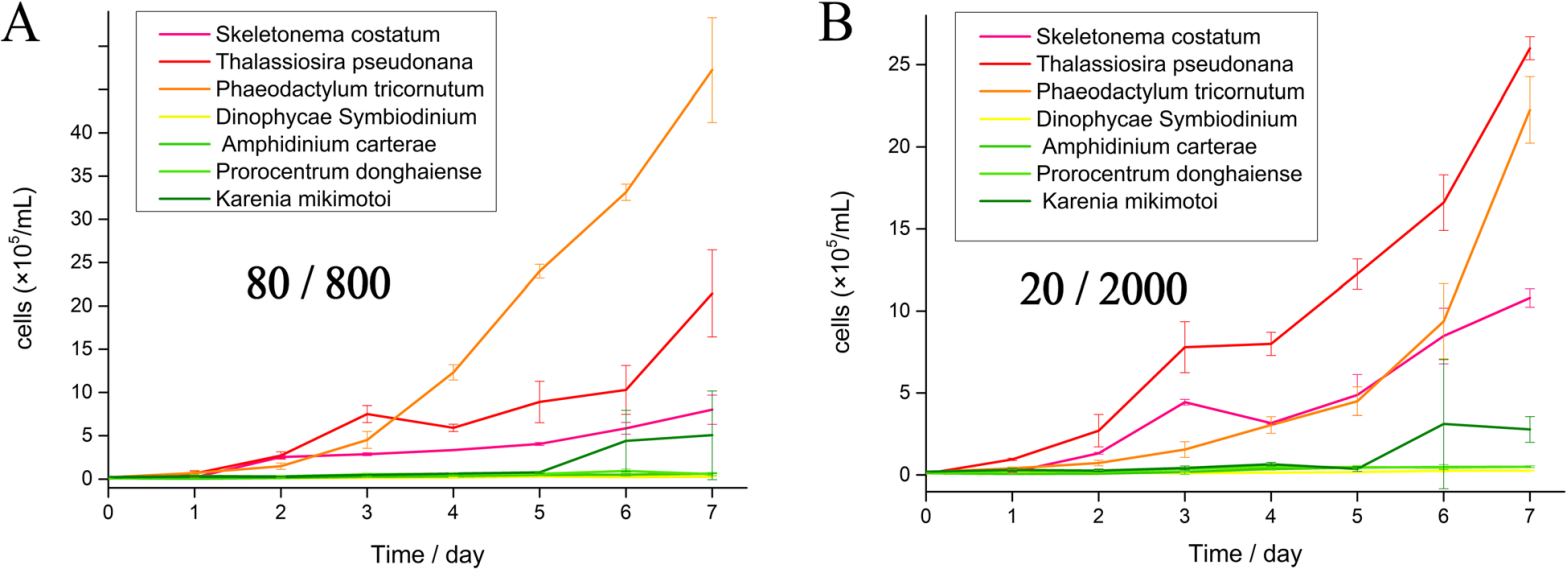
Growth curves of red tide diatom *S. costatum*, red tide dinoflagellate *A. carterae*, *P. donghaiense*, and *K. mikimotoi*, model diatom *P. tricornutum*, *T. pseudonana* and model dinoflagellate *D. Symbiodinium* under **a** mFL after the addition of 1 min of bright light (800 μmol photons m^− 2^ s^− 1^) to every 5 min of low light (80 μmol photons m^− 2^ s^− 1^) and **b** sFL after the addition of 1 min of stronger light (2000 μmol photons m^− 2^ s^− 1^) to every 5 min of low light (20 μmol photons m^− 2^ s^− 1^). Culture experiments were repeated three times

The light environment of the seawater surface during the upwelling season was simulated, and the growth rates of the marine diatom *P. tricornutum* and the dinoflagellates *P. donghaiense* and *K. mikimotoi*, were compared. Under both mFL and sFL, the three diatoms grew faster than the four dinoflagellates (Fig. 1).

**Fig. 2:**
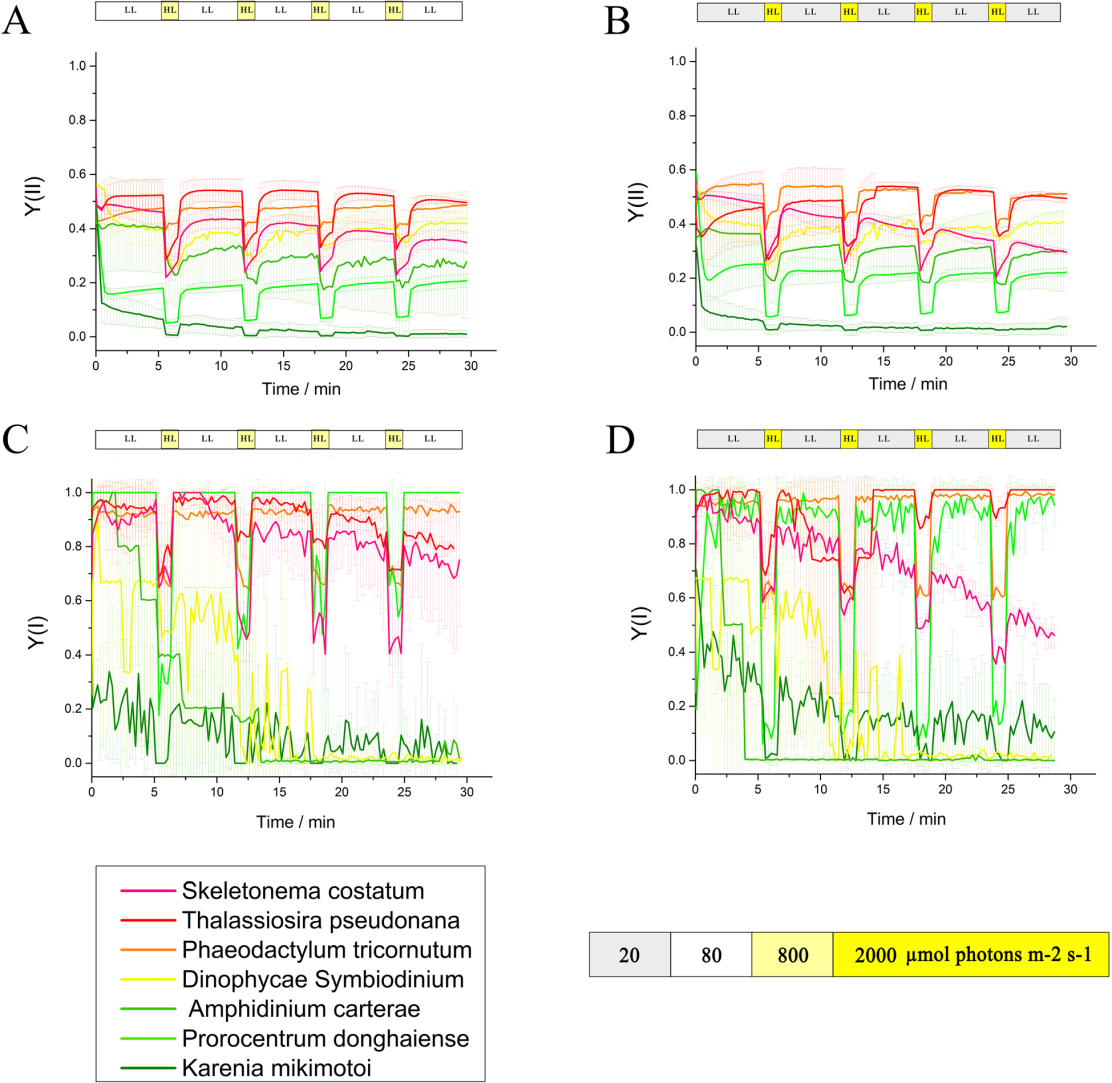
**a**, **b** Chlorophyll and **c**, **d** P700 fluorescence of red tide diatom *S. costatum*, red tide dinoflagellate *A. carterae*, *P. donghaiense*, and *K. mikimotoi*, model diatom *P. tricornutum*, *T. pseudonana* and model dinoflagellate *D. Symbiodinium* under **a**, **c** mFL after the addition of 1 min of bright light (800 μmol photons m^− 2^ s^− 1^) to every 5 min of low light (80 μmol photons m^− 2^ s^− 1^). **b**, **d** sFL after the addition of 1 min of stronger light (2000 μmol photons m^− 2^ s^− 1^) to every 5 min of low light (20 μmol photons m^− 2^ s^− 1^). The experiments have three replicates

To investigate the photosynthetic activities of these algae under dynamic light stress, PSI and PSII yields and related parameters were measured. Compared with those of the dinoflagellates, Y(I) and Y(II) of the diatoms showed smaller and more stable fluctuations with continuous conversion between strong and weak light. They also stayed active at a higher level when illuminated with sudden high light. After turning on the measured light, the Y(II) values of *P. donghaiense* and *K. mikimotoi* decreased rapidly and were less than 0.3 (Fig. 2). The Y(NA) of the three dinoflagellates increased erratically. Under mild light fluctuation, the Y(NA) values of the diatoms were lower than that of *K. mikimotoi*, whereas that of *P. donghaiense* was the lowest, although it had a high Y(ND) value (Fig. S1).

**Fig. 3:**
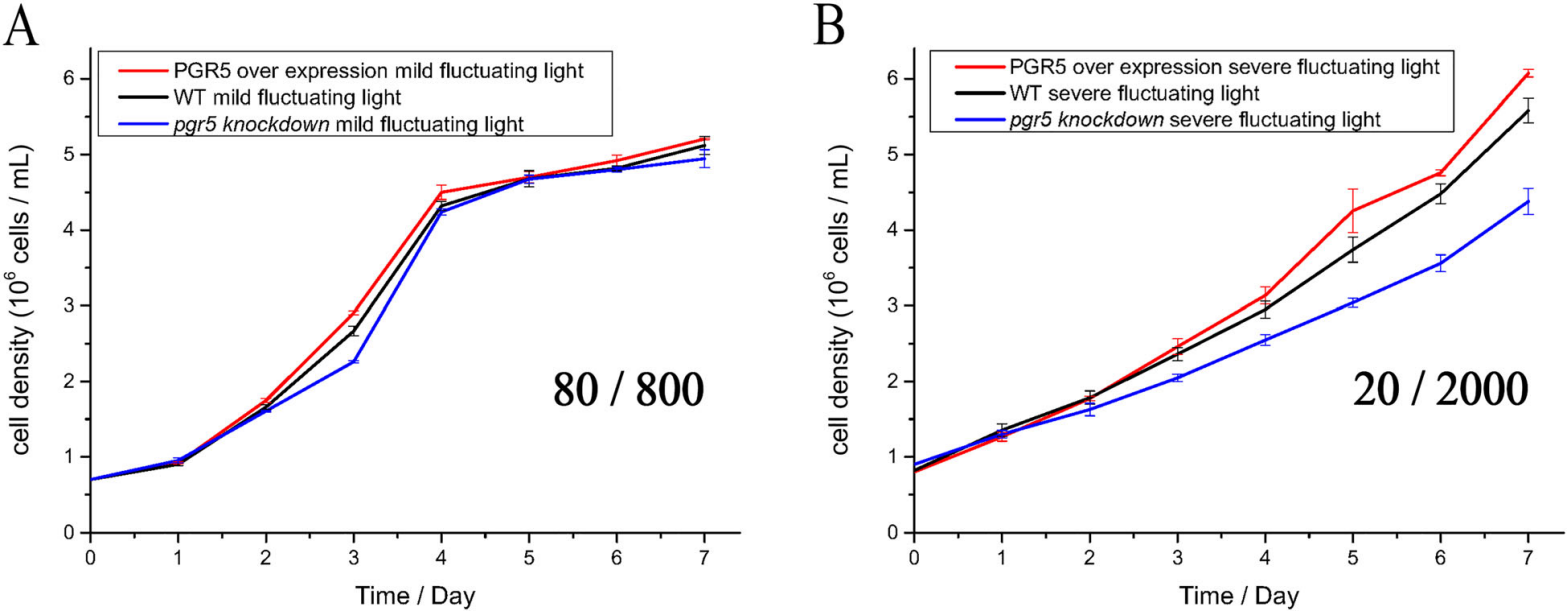
Fast kinetics of P700 during 300 ms SP after dark adaptation with or without 10 μM inhibitor Antimycin A (AA) in red tide diatom **b** *S. costatum*, red tide dinoflagellate **d** *A. carterae*, **e** *P. donghaiense*, and **f** *K. mikimotoi*, model diatom **c** *P. tricornutum*, **a** *T. pseudonana* and model dinoflagellate **g** *D. Symbiodinium*. Data was normalized to [0,1] using origin 9.0

The fast kinetics of P700 was measured under the first SP after dark adaptation, and AA was used to inhibit the PGR5/PGRL1-regulated CEF pathway. The P700 in the diatoms maintained a high oxidation level during SP and was sensitive to AA (Fig. 3, S1).

### Growth and photosynthetic parameters of PGR5 knocked down and overexpressing *P. tricornutum* under mFL and sFL

Since diatom was sensitive to the inhibitor AA, we used *P. tricornutum*, the model diatom with stable and efficient transformation methods, to investigate whether PGR5 in diatom responds to light fluctuation. Bioinformatics analysis revealed that the 61–132 base pair region that represents *PGR5* in *P. tricornutum* is conserved, and the first 20 bases form a potential signal peptide, as determined using SignalP-3.0 (http://www.cbs.dtu.dk/services/SignalP-3.0/). The PGR5 of Chlorophyta, Rhodophyta, Phaeophyta, and diatoms were on the same evolutionary branch as that of Cyanobacteria, but those of terrestrial green plants were on another branch. The diatom PGR5 was found to be closer to Phaeophyta and Rhodophyta PGR5. A protein similar to PGR5 was found in the dinoflagellates. However, several conserved sites were missing, especially when compared with those in motif 1, and no homology with sequences in other plants and algae was observed (Fig. S2).

We constructed 5OE-1 and 5KN-i1 and verified them using PCR amplification, quantitative PCR, and western blot analysis. The silencing or overexpression effect in the selected algal strain was significant (Fig. S3). In each cultivation treatment, *PGR5* overexpression and knockdown effects were maintained, but PGR5 expression was significantly higher under fluctuating light than under constant light (CL) condition.

**Fig. 4:**
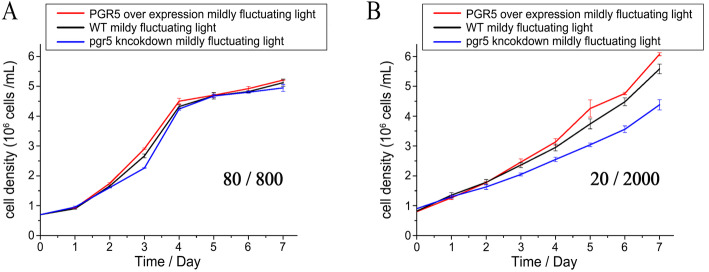
Growth curves under **a** mFL after the addition of 1 min of bright light (800 μmol photons m^− 2^ s^− 1^) to every 5 min of low light (80 μmol photons m^− 2^ s^− 1^) and **b** sFL after the addition of 1 min of stronger light (2000 μmol photons m^− 2^ s^− 1^) to every 5 min of low light (20 μmol photons m^− 2^ s^− 1^). Culture experiments were repeated three times. The values shown are the means of three biological replicates

The growth of *P. tricornutum* under fluctuating light was next investigated (Fig. 4). No differences were observed among the WT, 5OE-1, and 5KN-i1 under mFL. In contrast, under sFL, the growth rate of 5KN-i1 was significantly restricted, whereas that of 5OE-1 improved.

**Fig. 5:**
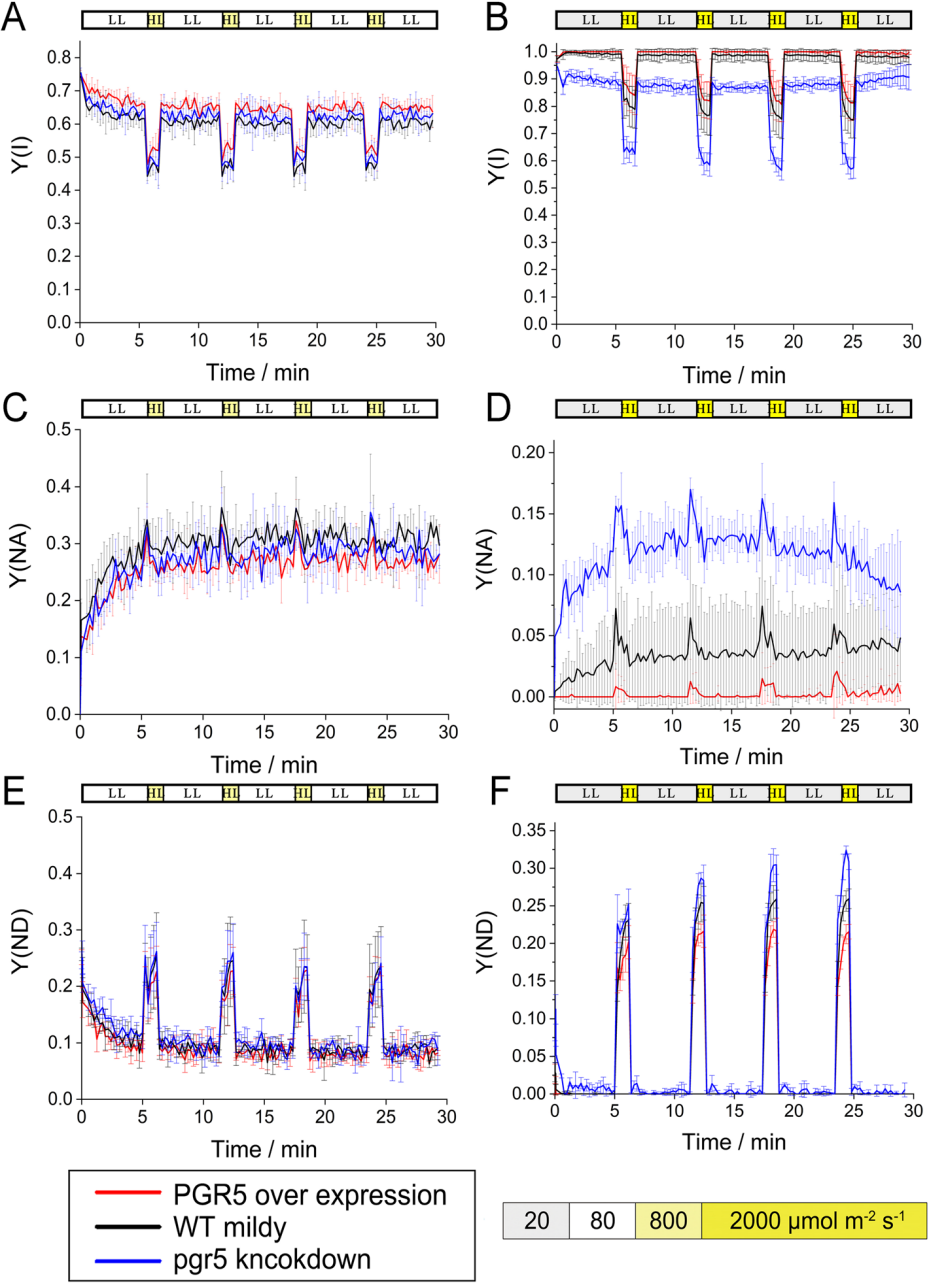
Photosynthetic parameters for P700. **a**, **c** and **e**: under mFL after the addition of 1 min of bright light (dark yellow, 800 μmol photons m^− 2^ s^− 1^) to every 5 min of low light (white, 80 μmol photons m^− 2^ s^− 1^); **b**, **d** and **f**: under sFL after the addition of 1 min of stronger light (bright yellow, 2000 μmol photons m^− 2^ s^− 1^) to every 5 min of low light (gray, 20 μmol photons m^− 2^ s^− 1^). The values shown are the means of at least three biological replicates. **a** and **b**: Y(I), PSI yield; **c** and **d**: Y(NA), PSI acceptor side limitation; **e** and **f**: Y(ND), PSI donor side limitation

The photosynthetic parameters of the WT, 5OE-1, and 5KN-i1 under mFL and sFL were measured. The P700 parameters for mFL and sFL differed significantly (Fig. 5). Under mFL, the PSI yields of WT, 5OE-1, and 5KN-i1 were similar, except for that of 5KN-i1, which increased slightly under bright light owing to increased donor side limitation. However, under sFL, the PSI acceptor side of 5KN-i1 was severely restricted, but the limitation in 5OE-1 was low. The differences in Y(NA) values between bright light and low light and Y(ND) values between dim or dark conditions and bright light showed the same trend.

**Fig. 6:**
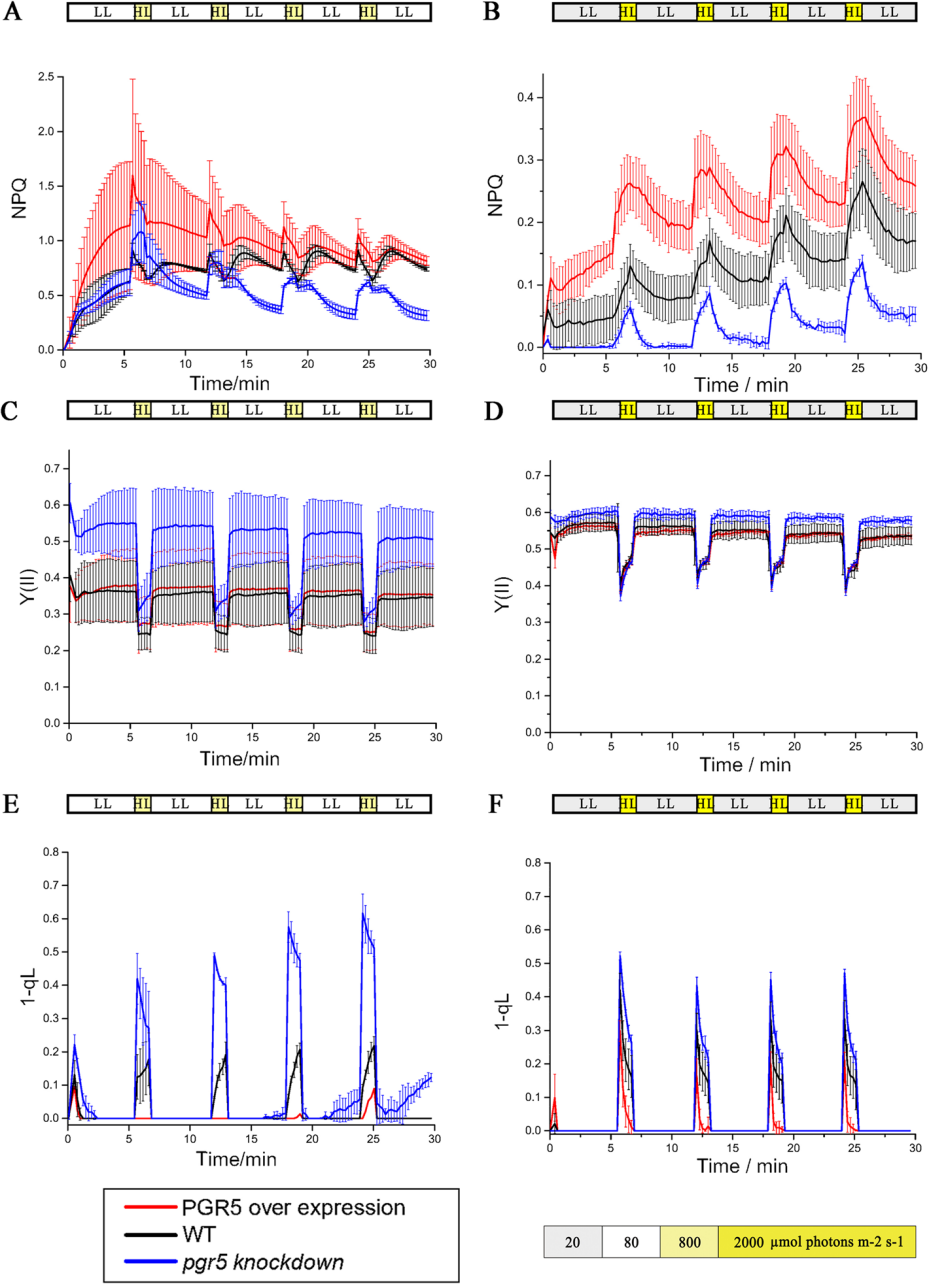
Photosynthetic parameters for PSII, **a**, **c** and **e**: under mFL after the addition of 1 min of bright light (dark yellow, 800 μmol photons m^− 2^ s^− 1^) to every 5 min of low light (white, 80 μmol photons m^− 2^ s^− 1^); and **b**, **d** and **f**: under sFL after the addition of 1 min of stronger light (bright yellow, 2000 μmol photons m^− 2^ s^− 1^) to every 5 min of low light (gray, 20 μmol photons m^− 2^ s^− 1^). The values shown are the means of at least three biological replicates. **a** and **b**: NPQ, non-photochemical quenching; **c** and **d**: Y(II), the actual conversion efficiency of light energy of PSII; **e** and **f**: 1 - qL, PQ redox state

Other photosynthetic characteristics also showed large differences among WT, 5OE-1, and 5KN-i1 (Fig. 6). The 1-qL and NPQ of 5KN-i1 decreased under the bright light phase of FL, whereas the Y(II) in 5KN-i1 increased under the low light phase of FL. In contrast, under high light, NPQ and 1-qL increased in 5OE-1. Under sFL, the differences in NPQ among WT, 5OE-1, and 5KN-i1 were more apparent, although the values were smaller overall. However, the difference in 1-qL was smaller under sFL. The Y(II) values also differed in that the Y(II) of 5KN-i1 was highest under mFL but equal to that in WT and 5OE-1 under sFL. Furthermore, the 1-qL in 5KN-i1 increased when moving from dark to low light under mFL and decreased in 5OE-1.

## Discussion

### Diatoms demonstrate better acclimation to fluctuating light than dinoflagellates, supporting diatom dominance in pioneer bloom of phytoplankton succession

Many researchers have focused on the succession of marine phytoplankton blooms. They recognized that depletion of nutrients, especially silicate, can cause diatom bloom recession and subsequent dinoflagellate bloom [9–11]. However, the key factor in early diatom blooms is still disputed [1, 2, 13–19, 21]. Researchers have attempted to identify factors that improve competitiveness in pioneer blooms and support huge biomass increases over several days in diatoms [1, 18, 28, 62].

In addition to nutrition, temperature, wind, and light were also investigated and are considered influential in pioneer blooms. However, all these traditional factors are contradictory and were regionally restricted in some marine surveys. Although the scale of dinoflagellates expands with increasing nitrogen or phosphate content, it cannot replace the pioneer role of the diatom [63–66]. Dinoflagellates can bloom in winter or quickly follow diatom blooms [2, 67–69], whereas dinoflagellate cysts may sprout slowly due to low temperatures when diatoms bloom. Increased temperature has the opposite effect in promoting bloom time or increasing predation pressure [2]. Sometimes, wind can reduce blooms [2], and light appears to lose its effect on the bloom. In fact, the mean value of light intensity does not represent the actual light environment well. In addition to environmental influences, differences in acclimation and growth between diatoms and dinoflagellates are also important. It is suggested that the higher growth rate of diatoms is the key to early blooms [1, 15, 18, 28, 68, 70]. Recently, some studies have suggested that diatoms have strong adaptation and recovery ability in long, cold, dark periods, which supports the bloom in late winter [71–73]. In fact, adaptation to complex and dynamic environments is also important in the large-scale spring bloom due to strong, coastal upwelling and violent waves without stabilized stratification.

**Fig. 7:**
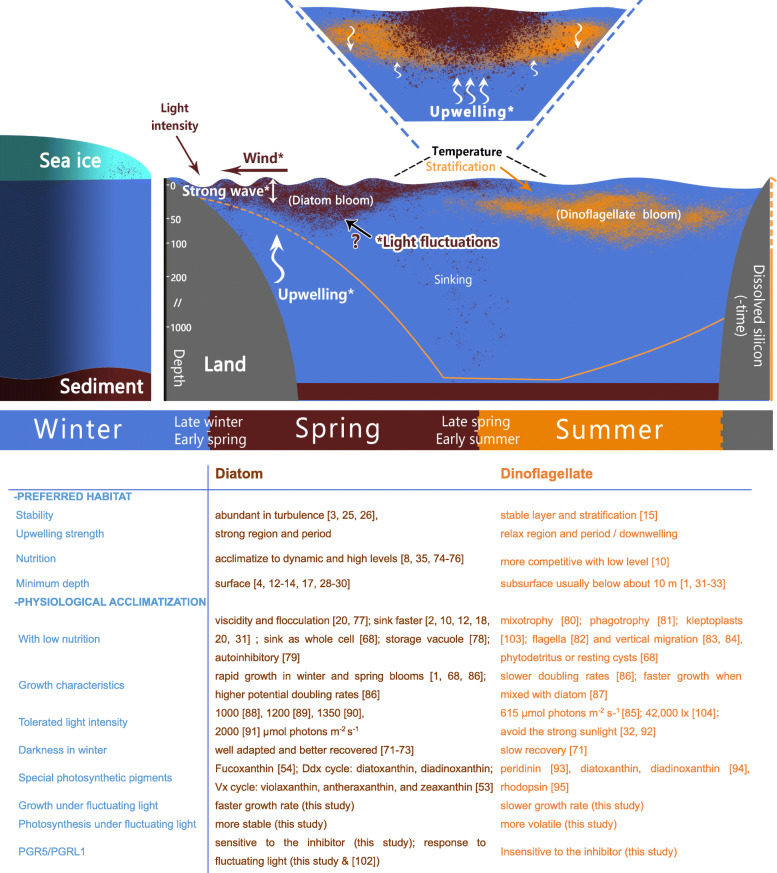
Traditional seasonal succession model of diatom and dinoflagellate blooms (not excluding the specificity of regional and annual differences and the impact of future climate change). The top part shows the distribution and environmental factors in diatom and dinoflagellate blooms. The bottom part lists currently known biological properties relative to the succession. The curve of dissolved silicon is referred to in previous work [10]. The graphic represents the monitored, decisive, non-biological factors in bloom succession (light, wind, and late silicate concentration) and the dotted line represents insufficient factors (temperature and early silicate concentration). Asterisk represents environmental factor associated to fluctuating light

Therefore, the current study suggests that light fluctuation and better acclimation to fluctuating light in diatoms, in addition to what is traditionally known, can also be the reasons for diatom dominance in pioneer blooms (Fig. 7 [74–99]). though acclimation to dynamic light in photosynthetic organisms on land and in fresh water has been studied in the past few years, the key mechanisms in marine algae are still unknown. The dominance of diatoms in a dynamic environment suggests potential mechanisms for adapting to fluctuating light, which confers an advantage over dinoflagellates that prefer a stable environment. With sufficient nutrients and without temperature limitations, diatoms have more growth advantages in the short term than dinoflagellates under both mild and severe light fluctuations (Fig. 1), which could support the fast bloom and absolute dominance of diatoms in a strong upwelling system or on the turbulent sea surface.

Photosynthesis is a key physiological process that plays an important role in the growth response to dynamic environmental stresses [56] Diatoms demonstrate more effective photosynthesis and better resistance under light fluctuation conditions than dinoflagellates (Fig. 2). In dynamic changes between bright and low light, the PSI and PSII of diatoms were able to stabilize these fluctuations and maintain high levels of activity. Diatoms use sudden periods of bright light more efficiently (even when saturated with sunlight [14, 100]), resulting in higher actual PSII and PSI yields than those in dinoflagellates. Efficient photosynthesis even under saturated illumination could support the huge biomass and sufficient acclimation on the sea surface. Even under sFL, the diatoms maintain lower PSI acceptor limitation (Fig. S1), indicating that they can more efficiently regulate excess electrons under fluctuating light, which affords them a stronger ability to resist and use fluctuating light to adapt to strong periods and regions of upwelling.

These results imply that light fluctuation can influence on the competition between diatoms and dinoflagellates.

### Photosynthesis can support rapid growth of pioneer diatoms under fluctuating light

The acclimation of photosynthesis to light fluctuations implies a molecular mechanism in diatoms that could promote growth. Diatoms were considered to lack FLV (an A-type flavoprotein), the key protein that acts in response to fluctuating light in freshwater algae [56, 101]. Notably, PGR5, another potentially critical protein in resisting light fluctuations, has been reported in the diatom proteome [120]. In this study, PGR5 was found to play an important role in the acclimation of diatoms to fluctuating light.

Antimycin A is an efficient and specific inhibitor of PGR5/PGRL1 depending on CEF. Previously, diatom CEF was measured to be low, and CEF (depending on PGR5/ PGRL1) was ignored in response to light fluctuation due to the moderate treatment [55]. Further, increased or decreased PGRL1 levels did not lead to enhancement of photosynthesis and growth in diatoms under fluctuating light [102]. The redox state of P700 was significantly lower after addition of AA to the diatoms than that in the dinoflagellates (Fig. 3), which implied that diatoms have a more effective CEF depending on PGR5/PGRL1, which is the key response to various light stresses. Although dinoflagellates contain the PGR5 homologous gene, it has no or negligible function, presumably due to abundant transferring genes in the mesokaryotic genome, a possible fusion gene with PGRL1 [103], or the minimally conserved C-terminal sequence (Fig. S2).

In the current study, PGR5 protein levels improved both photosynthetic electron flow and growth rate under sFL (between very low light [15] and saturated with sunlight [15, 100]). No significant growth differences were found under mild light fluctuation in 5KN-i1, WT, and 5OE-1 (Fig. 4a). However, under sFL, the growth rate of 5KN-i1 significantly decreased, whereas 5OE-1 grew rapidly. This implies that PGR5 in diatoms, which was underestimated previously, is sensitive to the intensity of light fluctuations. Photosynthesis showed the same result, especially in the acceptor side limitation of PSI [Y(NA)] (Fig. 5). In fact, PGR5 prevents PSI over-reduction on the acceptor side by increasing the production ratio of ATP/NADPH through ATP generation and electron transport for Cyt b6f or PQ. Compared with previous studies on the effect of dynamic light on terrestrial plants and freshwater algae, in this study, the special response to different light fluctuation intensities in the diatoms was associated with a preference for a concentrated bloom on the sea surface and a strong upwelling system. Indeed, diatoms always face frequent and large variations in light intensity due to shelter by high cell concentrations, wind agitation, and the surface lens effect. These results also reveal the crucial role of photosynthesis in diatom growth. Therefore, we suggest that the special acclimation mechanism of diatoms to fluctuating light might be imperative in their pioneering nature.

## Conclusion

This work implied that light fluctuation is a crucial factor in phytoplankton succession in diatoms and dinoflagellates. Diatoms are better acclimatized to fluctuating light than dinoflagellates in both growth and photosynthesis. Thus, diatoms acclimatize better to the dynamic environment and have growth advance in pioneer bloom under stronger upwelling, wave and wind conditions. PGR5 in diatom plays crucial roles under severe light fluctuation, supporting the photosynthesis adaptability and growth under variable environments of diatom. The results will also supplement to the responding mechanism to dynamic light in ocean photosynthesis organisms.

## Materials and methods

### Strain, cultivation, and growth monitoring

The diatom *Skeletonema costatum* and dinoflagellates *Amphidinium carterae*, *Prorocentrum donghaiense*, and *Karenia mikimotoi* are red tide species, whereas the diatoms *P. tricornutum* and *Thalassiosira pseudonana* and the dinoflagellate *Dinophycae Symbiodinium* are model species. Seven algae were grown in sterile artificial seawater containing F/2 medium at a temperature of 21 ± 1 °C. The light/dark cycle was set to 12 h/12 h. Following previous study on fluctuating light [56, 57, 101], mFL condition ranged from 100 to 10% of the bright light below the tolerance level of dinoflagellates (Fig. 7) [104]. In details, a 1-min period of bright light (800 μmol m^− 2^ s^− 1^) was added to each 5 min of low light (80 μmol m^− 2^ s^− 1^) in the mFL treatment. The larger fluctuating extent and higher light intensity were brought in sFL condition that ranged from 100% [14, 33, 34] to 1% [14, 15, 33] of the saturated sunlight. A 1 min of stronger light (2000 μmol m^− 2^ s^− 1^) was added to each 5 min of lower light (20 μmol m^− 2^ s^− 1^) in the sFL treatment.

The cell densities of the algae were counted using a hemocytometer, and growth of the wild-type (WT), *PGR5* overexpression algae (5OE-1), and *pgr5* knockdown algae (5KN-i1) of *P. tricornutum* was measured based on the absorbance at 730 nm wavelength (UV-1800, SHIMADZU, Japan). During the middle exponential growth phase, all the algae strains were centrifuged and flash-frozen at − 80 °C.

### Chlorophyll and P700 fluorescence parameter monitoring

During the exponential growth phase, the chlorophyll fluorescence parameters were monitored using a Dual-PAM-100 (Walz, Effeltrich, Germany) instrument with WinControl software 1 h after the beginning of the light cycle, under the same conditions used to culture the algae. The chlorophyll fluorescence and P700 were both measured using dual-channel equipment. The algae were kept in the dark for 10 min before measurement. Antimycin A (AA, 10 μM) was added when required [105]. The minimum fluorescence (F_0_), maximum fluorescence (F_m_, after a saturating flash), and maximum fluorescence (F_m_′, under photosynthetic active radiation) were measured and used to calculate the following values:

1 $$ \mathrm{Maximum}\ \mathrm{light}\ \mathrm{conversion}\ \mathrm{efficiency}:{\mathrm{F}}_{\mathrm{v}}/{\mathrm{F}}_{\mathrm{m}}=\left({\mathrm{F}}_{\mathrm{m}}-{\mathrm{F}}_0\right)/{\mathrm{F}}_{\mathrm{m}} $$

Effective PS II activity:

2 $$ \mathrm{Y}\left(\mathrm{II}\right)=\left(\mathrm{Fm}^{\prime }-\mathrm{F}\right)/\mathrm{Fm}^{\prime }, $$

3 $$ \mathrm{Non}-\mathrm{photochemical}\ \mathrm{quenching}:\mathrm{NPQ}=\left({\mathrm{F}}_{\mathrm{m}}-{\mathrm{F}}_{\mathrm{m}}\right)/{\mathrm{F}}_{\mathrm{m}}^{\prime }, $$

and Photochemical quenching coefficient:

4 $$ \mathrm{qL}=\left[\left({\mathrm{F}}_{\mathrm{m}}^{\prime }-\mathrm{F}\right)/\left({\mathrm{F}}_{\mathrm{m}}^{\prime }-{\mathrm{F}}_{\mathrm{o}}^{\prime}\right)\cdotp {\mathrm{F}}_{\mathrm{o}}^{\prime }/\mathrm{F}\right]. $$

Similarly, the maximum change in the P700 signal (P_m_), maximum change in the P700 signal under photosynthetic active radiation (P_m_′), zero P700 signal (P_0_), and the fraction of overall P700 reduction after the saturation pulse were used to calculate the photosystem I (PSI) donor side limitation (Y[ND]), acceptor side limitation (Y[NA]), and the PSI yield, using the following equation:

5 $$ \mathrm{Y}\left(\mathrm{I}\right)=1-\mathrm{Y}\left(\mathrm{ND}\right)-\mathrm{Y}\left(\mathrm{NA}\right) $$

### Phylogenetic analysis and transformation

The *PGR5* sequence in *P. tricornutum* (Gene ID: 7199723) was downloaded from the National Center for Biotechnology Information database. Protein sequences of other species were downloaded from the National Center for Biotechnology Information and UNIPROT databases. Similar sequences of *PGR5* in dinoflagellates were identified using NCBI-BLAST [106] from the genome assembly (WGS Project: BASF01, BGNK01, BGPT01, GAFO01, GBSC01, GFLM01, GFPM01, GHKS01, GICE01, IADN01, IADM01, VSDK01) and RNA-seq of *Prorocentrum donghaiense* (Accession: PRJNA374496) [107–116]. MEGA 7.0 was used to construct a maximum likelihood evolutionary tree after alignment using ClustalW.

A 157 bp partial sequence containing motif 4 (which is predicted, using MEME suite 5.1.1 [117], to be unique to diatoms) and the 3′ untranslated region (UTR) of *P. tricornutum* were amplified, and the products were digested using HindIII (sense) and EcoRI (anti-sense). The sequence was inserted in reverse into the multiple cloning site of the pPha-T1 vector [118] (after FcpA promoter, a strong promoter resounding to light [119]) using EcoRI and HindIII restriction enzyme digestion for *PGR5* knockdown in *P. tricornutum*. Vectors for *PGR5* overexpression were generated by cloning the full-length *PGR5* gene and inserting it in the forward direction.

Gold particles (50 μL/3 mg) with 20 μL spermidine (0.1 M), 50 μL CaCl_2_ (2.5 M) and 5 μg plasmids (approximately 10 μL) were used for transformation using particle bombardment (BIO-RAD Biolistic PDS-1000/H2 Particle Delivery System, CA, USA) [51, 61]. After 24 h of recovery under low light, the algae were transferred onto plates containing zeocin (100 μg/mL). After 20–30 days, individual algal colonies were retrieved and lysed for direct polymerase chain reaction (PCR) to verify sequence integration. The individual colonies were then cultured in liquid medium containing zeocin (100 μg/mL).

### Real-time PCR analysis

Real-time PCR was used to verify that the target sequence had been integrated into the genome and to determine the *PGR5* mRNA expression level (Fig. S3). RNA extraction and real-time PCR analysis were performed according to methods described by Wu et al. [105]. Total RNA was obtained from frozen algae using an RNA prep Pure Plant kit (polysaccharide and polyphenolic-rich) (Tiangen, Beijing, China). RNA quality was assessed using a NanoPhotometer (IMPLEN, Munich, Germany) and 1% agarose gel electrophoresis. Reverse transcription was performed using a PrimeScript RT reagent kit with gRNA Eraser (Takara, Beijing, China). The cDNA was used as a template and quantified via quantitative PCR, using a FastStart Essential DNA Green Master (Roche) in an iQ5 multicolor real-time PCR detection system (Bio-Rad, Hercules, USA) with Bio-Rad optical system software. The internal control was the RPS (ribosomal protein small subunit 30S) gene (Table S1).

### Sodium dodecyl sulfate-polyacrylamide gel electrophoresis and immunoblotting analysis

Protein levels were quantified using western blot analysis. Thylakoid membrane protein extraction was performed according to the method described by Grouneva et al. [120]. Frozen algae (collected from fresh algae approximately in the middle of their exponential growth period) were ground in liquid nitrogen and centrifuged at 300×*g* to remove cell debris and impurities after the addition of extraction buffer (10 mM MES, 2 mM KCl, 5 mM Na_2_EDTA, and 1 M D-sorbitol; pH 6.5). The sediment was ground again in extraction buffer and centrifuged at 300×*g*. All supernatants were collected and centrifuged at 20,000×*g*. The sediment was washed twice in lysis buffer (which was identical to the extraction buffer, except that it did not contain d-sorbitol) and then suspended in storage solution (25 mM Tris-HCl and 20% glycerol; pH 7.0). All solutions were pre-cooled at 4 °C, and the thylakoid membrane proteins were stored at − 80 °C. The chlorophyll and BCA methods (Fig. S3) were used to quantify the protein, and 5 μg of Chl or 50 μg of protein (for pigment change under different conditions) was analyzed using sodium dodecyl sulfate-polyacrylamide gel electrophoresis [121, 122]. Antibodies against PsaB (photosystem I P700 chlorophyll a apoprotein A2) and ATPB (ATP synthase subunit β, chloroplastic) were purchased from Agrisera (Vännäs, Sweden), and an antibody to PGR5 was produced in our laboratory (antigenic peptide sequence: TKLIKKAKVNGDTLGF).

## Supplementary Information

**Additional file 1: Figure S1.** Y(NA) (PSI acceptor side limitation), Y(ND) (PSI donor side limitation), and 1–qL (PQ redox state) of red tide diatom *S. costatum*, red tide dinoflagellate *A. carterae*, *P. donghaiense*, and *K. mikimotoi*, model diatom *P. tricornutum*, *T. pseudonana* and model dinoflagellate *D. Symbiodinium* under (**A**) mild light fluctuation after the addition of 1 min of bright light (800 μmol photons m^-2^ s^-1^) to every 5 min of low light (80 μmol photons m^-2^ s^-1^) and (**B**) severe light fluctuation after the addition of 1 min of stronger light (2,000 μmol photons m^-2^ s^-1^) to every 5 min of low light (20 μmol photons m^-2^ s^-1^).

**Additional file 2: Figure S2.** Conserved sequences in, and evolutionary relationships of, PGR5 in plants and algae. Known signal peptide sequences are underlined. Motifs were identified using the MEME motif elicitation tool (Version 5.0.5). The missing conserved sites of dinoflagellates are shown with a red cross. The sequences are available in NCBI database (http://www.ncbi.nlm.nih.gov) or UniProtKB/TrEMBL database (https://www.uniprot.org/) as following. Eudicots: Arabidopsis thaliana (gi: 330250863), Cucumis sativus (gi: 164449273), Artemisia annua (gi: 1387830212), Gossypium arboreum (tr: I1ZIR9); Amborella: Amborella trichopoda (gi: 586688763); Gymnosperm: Pinus taeda (gi: 196168724); monocots: Triticum aestivum (gi: 393690734), Brachypodium distachyon (gi: 357144276), Oryza brachyantha (gi: 573956334), Panicum hallii (gi: 1435170242), Zea mays (gi: 1394909989); Mosses: Physcomitrella patens (gi: 1373914553); Klebsormidiales: Klebsormidium nitens (gi: 971519293); Chlorophyta: Tetrabaena socialis (gi: 1331346858), Monoraphidium neglectum (gi: 926775414), Auxenochlorella protothecoides (gi: 675355490); Cyanobacteria: Acaryochloris marina MBIC11017 (gi: 158308814), Cyanothece sp. PCC 7425 (gi: 219867356); Rhodophyta: Porphyra umbilicalis (gi: 1189386569), Pyropia yezoensis (tr: A1YSQ5), Gracilariopsis chorda (gi: 1395913517), Phaeophyta: Ectocarpus siliculosus (tr: D7G229); Diatom: Thalassiosira pseudonana (tr: B8C035), Phaeodactylum tricornutum (strain CCAP 1055/1) (tr: B7FVH9); Dinoflagellates: sequences were identified using NCBI-BLAST (BASF01, BGNK01, BGPT01, GAFO01, GBSC01, GFLM01, GFPM01, GHKS01, GICE01, IADN01, IADM01, VSDK0, PRJNA374496).

**Additional file 3: Figure S3.** Relative (**A**) RNA levels of PGR5 under 80 μmol photons m^-2^ s^-1^ and (**B**) thylakoid membrane protein levels (PGR5, PsaB, and AtpB) in the WT, 5OE-1, and 5KN-i1 under different light conditions. The protein was quantified using the BCA method (50 μg protein, ~3 μg Chl). CL: constant light under 80 μmol photons m^-2^ s^-1^; mFL: mildly fluctuating light: addition of 1 min of bright light (800 μmol photons m^-2^ s^-1^) to every 5 min of low light (80 μmol photons m^-2^ s^-1^); sFL: severely fluctuating light: addition of 1 min of stronger light (2,000 μmol photons m^-2^ s^-1^) to every 5 min of low light (20 μmol photons m^-2^ s^-1^). ANOVA was calculated by SPSS 23.0 (P<0.01).

**Additional file 4: Table S1.**. Primers used in transgene construction. The restriction sites are shown in bold.

## Data Availability

The datasets used and/or analyzed during the current study are available from corresponding authors on reasonable request. The sequences used in this work are available in NCBI database (http://www.ncbi.nlm.nih.gov) or UniProtKB/TrEMBL database (https://www.uniprot.org/). NCBI accession numbers are 330,250,863, 164,449,273, 1,387,830,212, 586,688,763, 196,168,724, 393,690,734, 357,144,276, 573,956,334, 1,435,170,242, 1,394,909,989, 1,373,914,553, 971,519,293, 1,331,346,858, 926,775,414, 675,355,490, 158,308,814, 219,867,356, 1,189,386,569, 1,395,913,517; UniProtKB/TrEMBL identifiers are I1ZIR9, A1YSQ5, D7G229, B8C035, B7FVH9; NCBI-Assembly accession numbers are BASF01, BGNK01, BGPT01, GAFO01, GBSC01, GFLM01, GFPM01, GHKS01, GICE01, IADN01, IADM01, VSDK01,PRJNA374496.

## Abbreviations

PGR5: Proton Gradient Regulation 5
mFL: mildly fluctuating light
sFL: severely fluctuating light
CL: constant light
WT: wild-type
5OE-1: PGR5 overexpression algae
5KN-i1: pgr5 knockdown algae
AA: Antimycin A
CEF: cyclic electron flow
FLV: an A-type flavoprotein)
Y(I): PSI yield
Y(NA): PSI acceptor side limitation
Y(ND): PSI donor side limitation
NPQ: non-photochemical quenching
Y(II): the actual conversion efficiency of light energy of PSII
1–qL: PQ redox state
