# Correction to: Photosynthesis acclimation under severely fluctuating light conditions allows faster growth of diatoms compared with dinoflagellates

**DOI:** 10.1186/s12870-021-02984-w

**Published:** 2021-04-30

**Authors:** Lu Zhou, Songcui Wu, Wenhui Gu, Lijun Wang, Jing Wang, Shan Gao, Guangce Wang

**Affiliations:** 1 CAS and Shandong Province Key Laboratory of Experimental Marine Biology, Center for Ocean Mega-Science, Institute of Oceanology, Chinese Academy of Sciences, Qingdao, 266071 China; 2 Laboratory for Marine Biology and Biotechnology, Qingdao National Laboratory for Marine Science and Technology, Qingdao, 266237 China; 3 College of Earth Sciences, University of Chinese Academy of Sciences, Beijing, 100049 China

**Correction to: BMC Plant Biol 21, 164 (2021)**

**https://doi.org/10.1186/s12870-021-02902-0**

Following publication of the original article [1], the authors identified that Fig. 3 appears identical with Fig. 4. In fact, the Fig. 3 has never been changed from the original submitted manuscript to the revision and proof process. The order and legend of Fig. 3 were also not changed in the submission and revision. The loss of Fig. 3 was due to the careless manipulation during the typesetting process. The correct Fig. 3 is provided below:

**Fig. 3:**
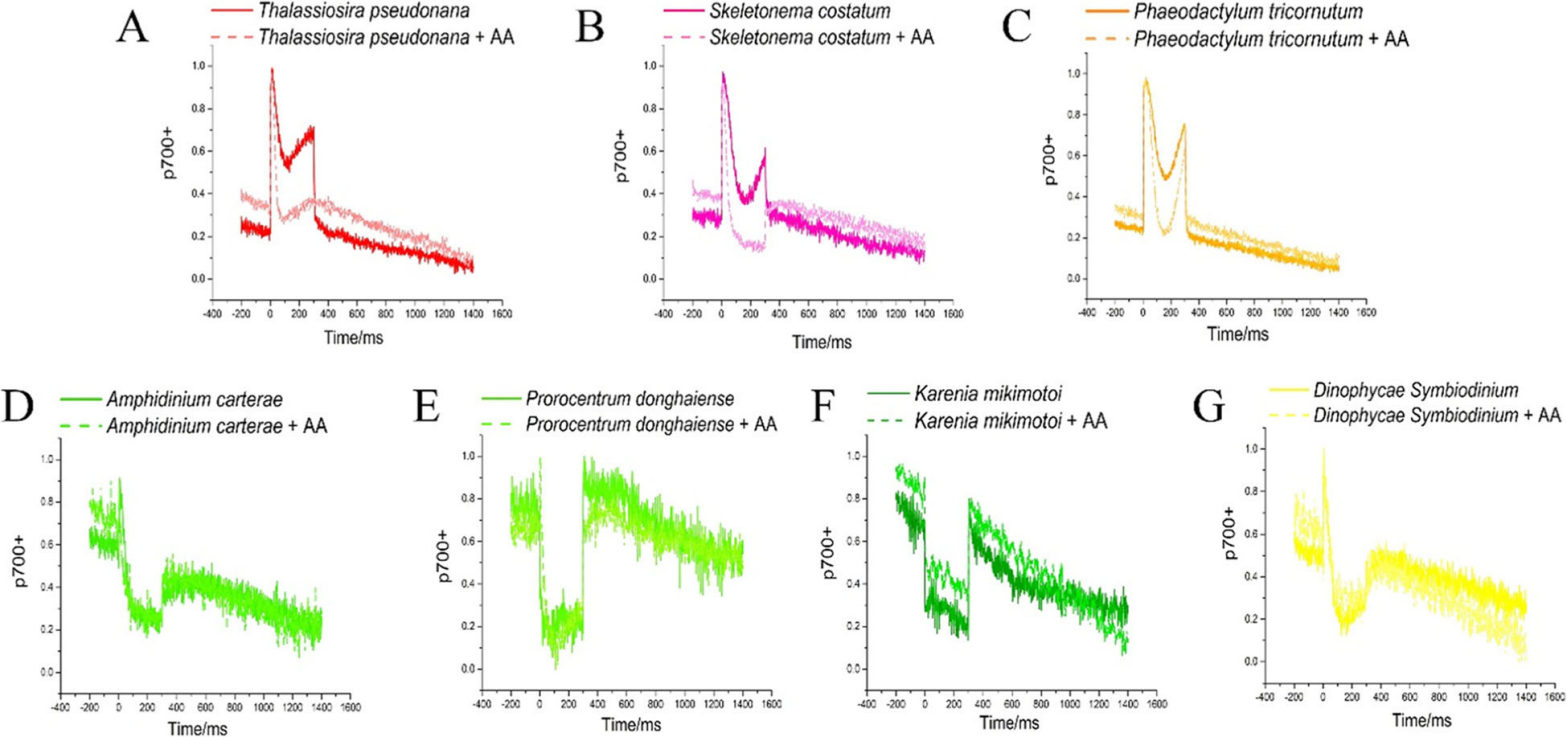
Fast kinetics of P700 during 300 ms SP after dark adaptation with or without 10 μM inhibitor Antimycin A (AA) in red tide diatom **b** *S. costatum*, red tide dinoflagellate **d** *A. carterae*, **e** *P. donghaiense*, and **f** *K. mikimotoi*, model diatom **c** *P. tricornutum*, **a** *T. pseudonana* and model dinoflagellate **g** *D. Symbiodinium*. Data was normalized to [0,1] using origin 9.0

The correction does not have any effect on the results or conclusions of the paper. The original article has been corrected.
