# Supplementary figures and images for: Photosynthesis acclimation under severely fluctuating light conditions allows faster growth of diatoms compared with dinoflagellates
Source: BMC Plant Biol. 2021 Apr 1;21:164. doi: 10.1186/s12870-021-02902-0 (PMC8015109; doi:10.1186/s12870-021-02902-0)

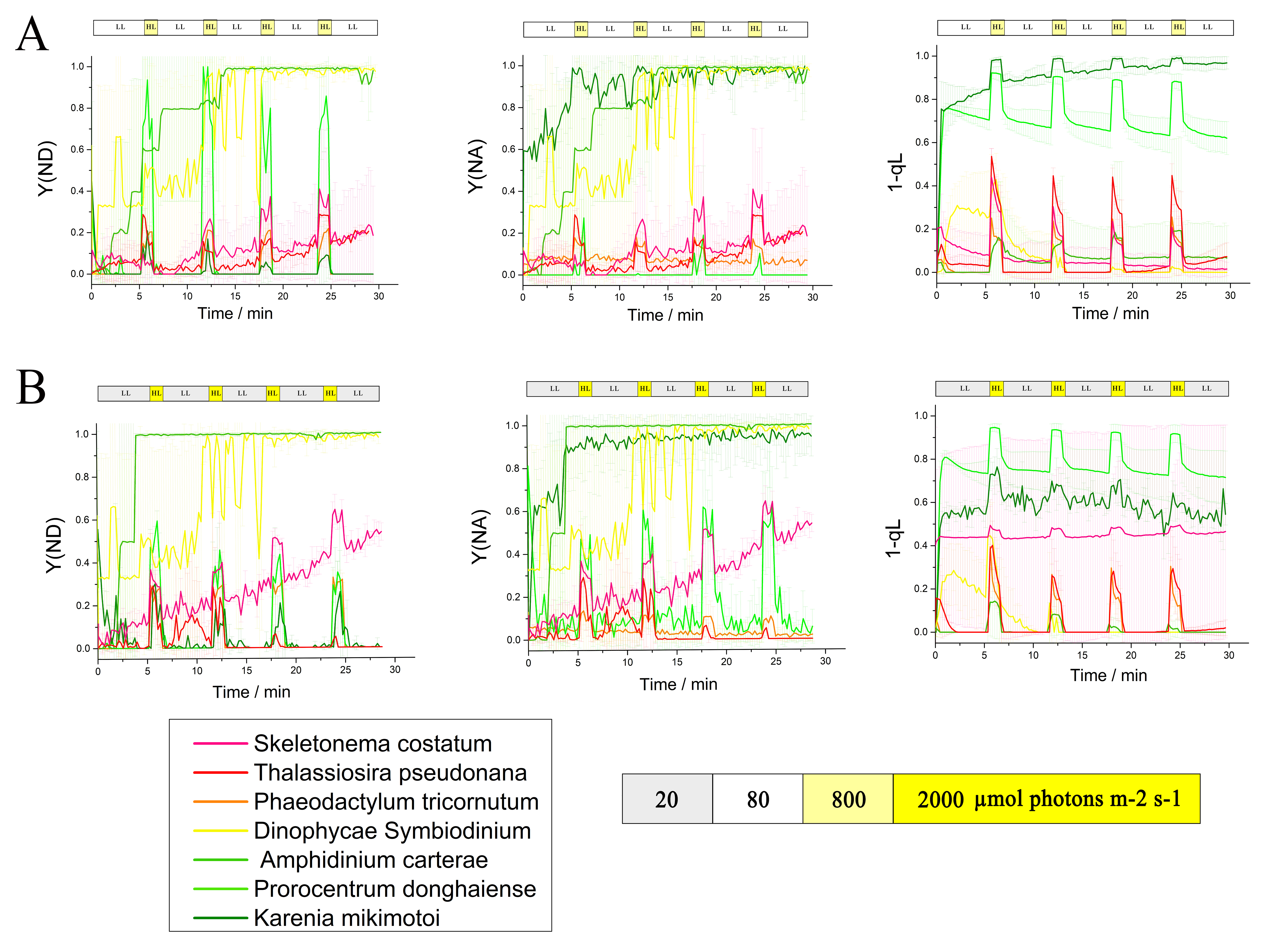

Supplement: Supplementary file 1 — Additional file 1: Figure S1. Y(NA) (PSI acceptor side limitation), Y(ND) (PSI donor side limitation), and 1–qL (PQ redox state) of red tide diatom S. costatum, red tide dinoflagellate A. carterae, P. donghaiense, and K. mikimotoi, model diatom P. tricornutum, T. pseudonana and model dinoflagellate D. Symbiodinium under (A) mild light fluctuation after the addition of 1 min of bright light (800 μmol photons m-2 s-1) to every 5 min of low light (80 μmol photons m-2 s-1) and (B) severe light fluctuation after the addition of 1 min of stronger light (2,000 μmol photons m-2 s-1) to every 5 min of low light (20 μmol photons m-2 s-1). [file 12870_2021_2902_MOESM1_ESM.jpg]

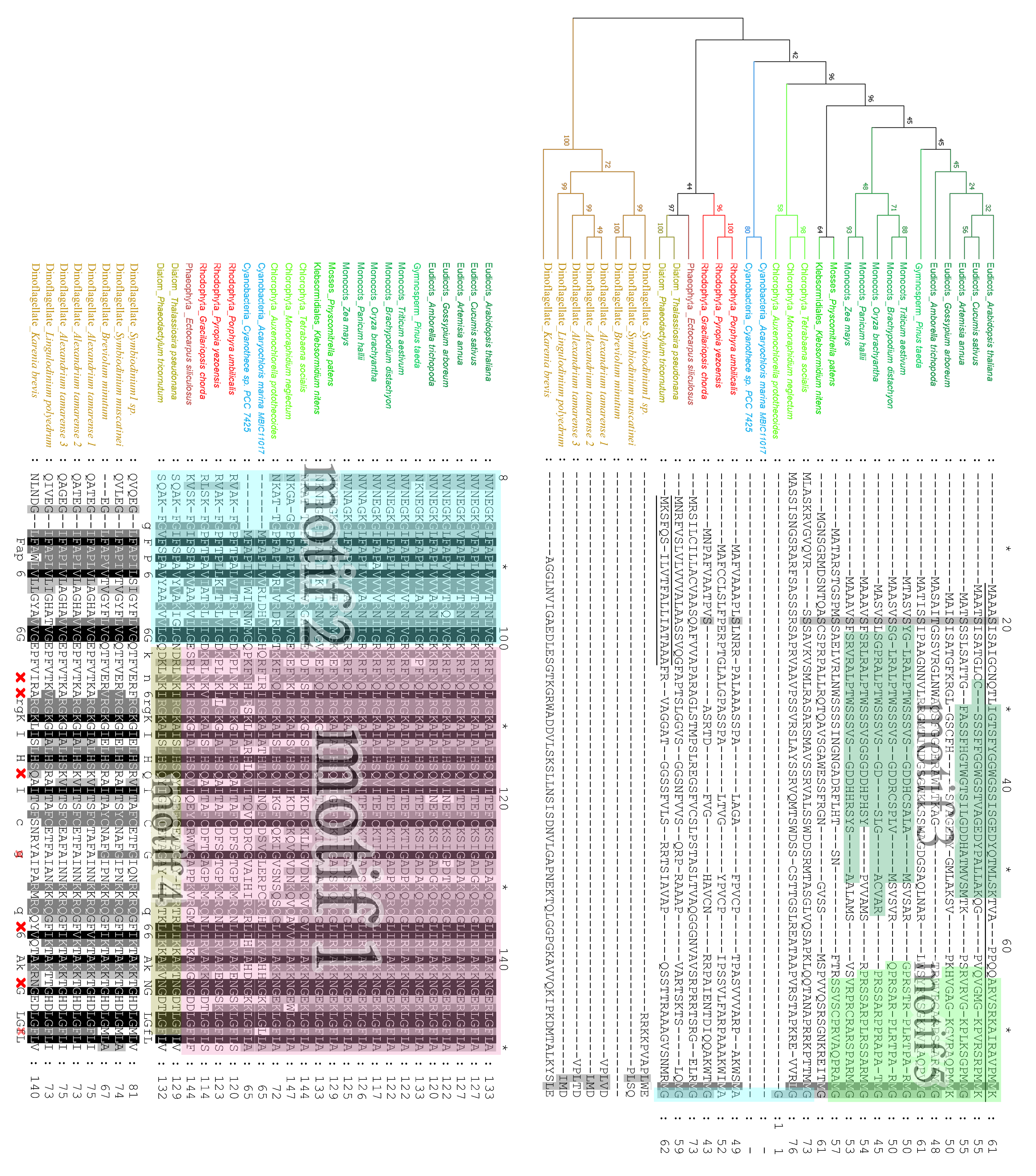

Supplement: Supplementary file 2 — Additional file 2: Figure S2. Conserved sequences in, and evolutionary relationships of, PGR5 in plants and algae. Known signal peptide sequences are underlined. Motifs were identified using the MEME motif elicitation tool (Version 5.0.5). The missing conserved sites of dinoflagellates are shown with a red cross. The sequences are available in NCBI database (http://www.ncbi.nlm.nih.gov) or UniProtKB/TrEMBL database (https://www.uniprot.org/) as following. Eudicots: Arabidopsis thaliana (gi: 330250863), Cucumis sativus (gi: 164449273), Artemisia annua (gi: 1387830212), Gossypium arboreum (tr: I1ZIR9); Amborella: Amborella trichopoda (gi: 586688763); Gymnosperm: Pinus taeda (gi: 196168724); monocots: Triticum aestivum (gi: 393690734), Brachypodium distachyon (gi: 357144276), Oryza brachyantha (gi: 573956334), Panicum hallii (gi: 1435170242), Zea mays (gi: 1394909989); Mosses: Physcomitrella patens (gi: 1373914553); Klebsormidiales: Klebsormidium nitens (gi: 971519293); Chlorophyta: Tetrabaena socialis (gi: 1331346858), Monoraphidium neglectum (gi: 926775414), Auxenochlorella protothecoides (gi: 675355490); Cyanobacteria: Acaryochloris marina MBIC11017 (gi: 158308814), Cyanothece sp. PCC 7425 (gi: 219867356); Rhodophyta: Porphyra umbilicalis (gi: 1189386569), Pyropia yezoensis (tr: A1YSQ5), Gracilariopsis chorda (gi: 1395913517), Phaeophyta: Ectocarpus siliculosus (tr: D7G229); Diatom: Thalassiosira pseudonana (tr: B8C035), Phaeodactylum tricornutum (strain CCAP 1055/1) (tr: B7FVH9); Dinoflagellates: sequences were identified using NCBI-BLAST (BASF01, BGNK01, BGPT01, GAFO01, GBSC01, GFLM01, GFPM01, GHKS01, GICE01, IADN01, IADM01, VSDK0, PRJNA374496). [file 12870_2021_2902_MOESM2_ESM.jpg]

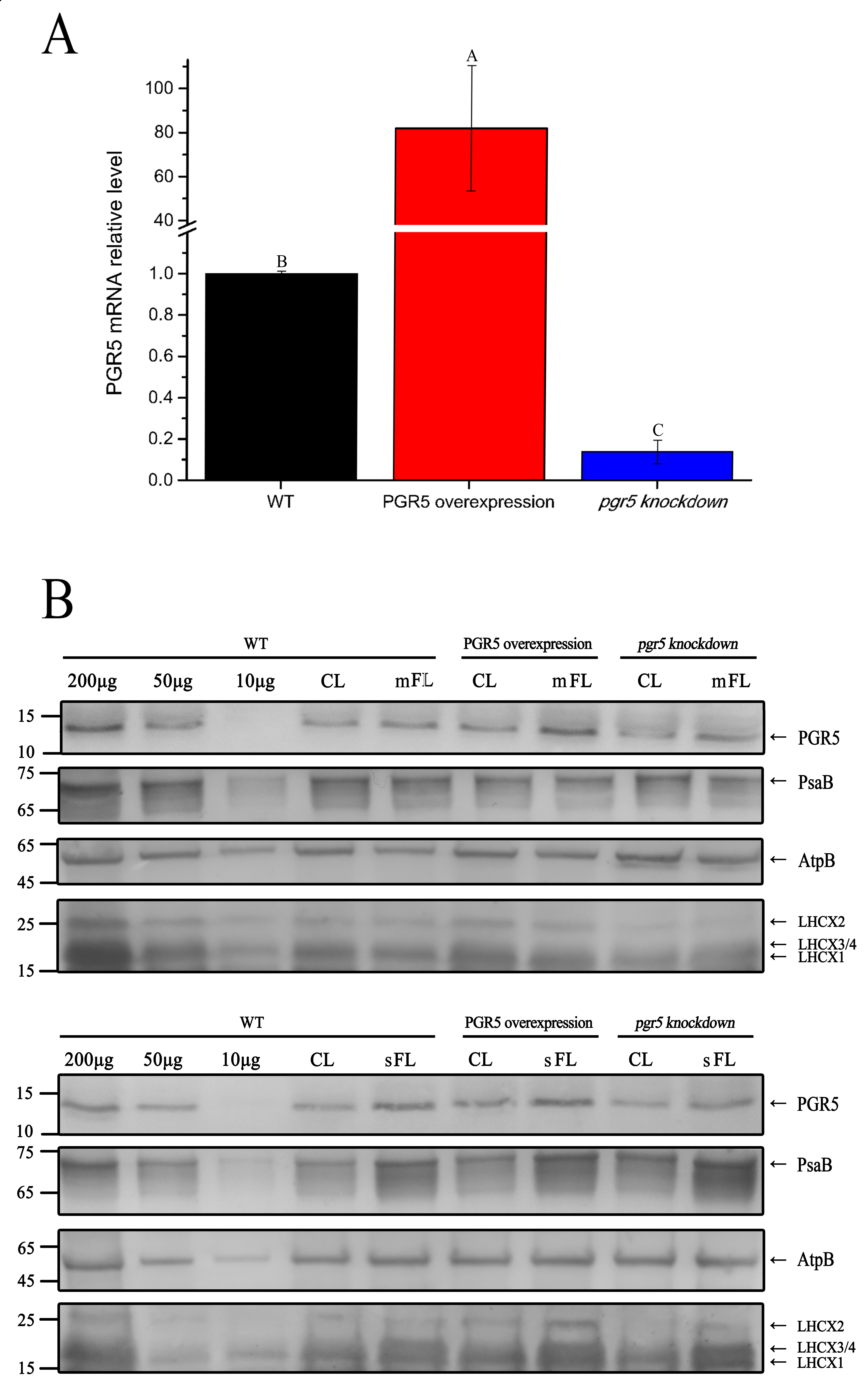

Supplement: Supplementary file 3 — Additional file 3: Figure S3. Relative (A) RNA levels of PGR5 under 80 μmol photons m-2 s-1 and (B) thylakoid membrane protein levels (PGR5, PsaB, and AtpB) in the WT, 5OE-1, and 5KN-i1 under different light conditions. The protein was quantified using the BCA method (50 μg protein, ~3 μg Chl). CL: constant light under 80 μmol photons m-2 s-1; mFL: mildly fluctuating light: addition of 1 min of bright light (800 μmol photons m-2 s-1) to every 5 min of low light (80 μmol photons m-2 s-1); sFL: severely fluctuating light: addition of 1 min of stronger light (2,000 μmol photons m-2 s-1) to every 5 min of low light (20 μmol photons m-2 s-1). ANOVA was calculated by SPSS 23.0 (P<0.01). [file 12870_2021_2902_MOESM3_ESM.jpg]
