# Supplementary material for: Photosynthesis acclimation under severely fluctuating light conditions allows faster growth of diatoms compared with dinoflagellates
Source: BMC Plant Biol. 2021 Apr 1;21:164. doi: 10.1186/s12870-021-02902-0 (PMC8015109; doi:10.1186/s12870-021-02902-0)
Supplement: Supplementary file 4 — Additional file 4: Table S1.. Primers used in transgene construction. The restriction sites are shown in bold. [file 12870_2021_2902_MOESM4_ESM.docx]

**Photosynthesis acclimation under severely fluctuating light conditions allows faster growth of diatoms compared with dinoflagellates**

Lu Zhou ^a, b, c, d^, Songcui Wu ^a, b, d^, Wenhui Gu ^a, b, d^, Lijun Wang ^a, b, d^, Jing Wang ^a, b, d^, Shan Gao ^*, a, b, d^ & Guangce Wang ^*, a, b, d^

a Key Laboratory of Experimental Marine Biology, Institute of Oceanology, Chinese Academy of Sciences, Qingdao 266071, China;

b Laboratory for Marine Biology and Biotechnology, Qingdao National Laboratory for Marine Science and Technology, Qingdao 266237, China;

c College of Earth Sciences, University of Chinese Academy of Sciences, Beijing 100049, China;

d Center for Ocean Mega-Science, Chinese Academy of Sciences, 7 Nanhai Road, Qingdao, 266071, P. R. China;

***Corresponding author:**

Guangce Wang, E-mail, gcwang@qdio.ac.cn; Fax, +86-532-82880645;

Shan Gao, E-mail, shangao@qdio.ac.cn; Fax, +86-532-82880645.

Supplemental Materials

**Table S1**. Primers used in transgene construction. The restriction sites are shown in bold.

|  | **Primer Sequence (5′-3′)** |
| --- | --- |
| **RPS (ribosomal protein small subunit 30S)** | Sense: CGAAGTCAACCAGGAAACCAA  Anti-sense: GTGCAAGAGACCGGACATACC |
| ***pgr5 knockdown*** | Sense: **GAATTC**CTACCACCTACGAACCAAA  Anti-sense: **AAGCTT**AAGACGGGAGAAACAGACAA |
| **PGR5 overexpression** | Sense: **GAATTC**ACGAACTGTCTACCGATTCC  Anti-sense: **AAGCTT**CAGCAAATGATAACGGATGG |
| **Direct PCR** | Sense: GGAGGATCAGATTCAGATTACA  Anti-sense: AGAGCAAGGATGCCCATT |
| **qPCR of PGR5** | Sense: GGGGTTCTTCCTTTGTGCT  Anti-sense: CGACTTTGGCGGCGTA |


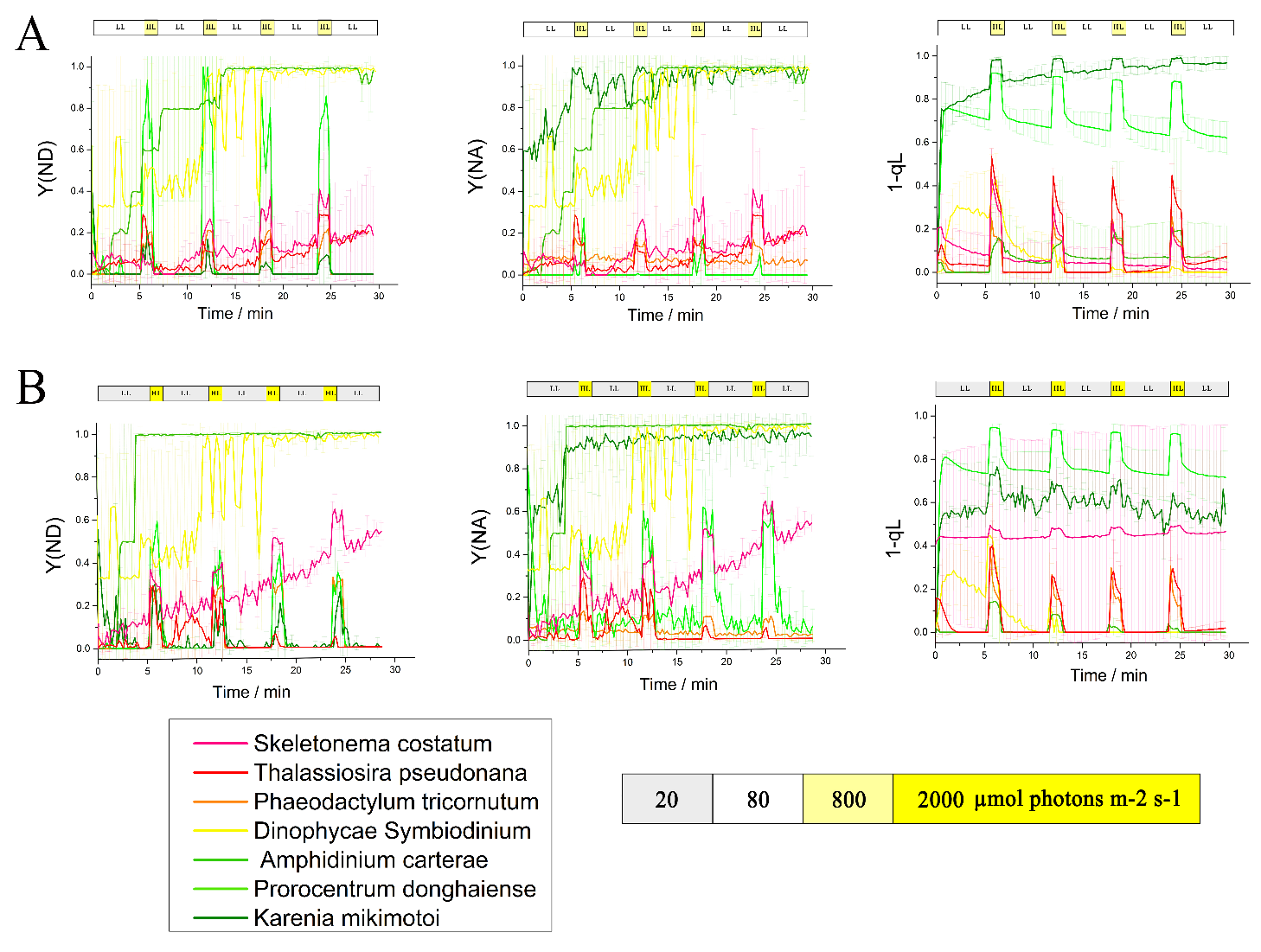


**Figure S1** Y(NA) (PSI acceptor side limitation), Y(ND) (PSI donor side limitation), and 1–qL (PQ redox state) of red tide diatom *S. costatum*, red tide dinoflagellate *A. carterae*, *P. donghaiense*, and *K. mikimotoi*, model diatom *P. tricornutum*, *T. pseudonana* and model dinoflagellate *D. Symbiodinium* under (**A**) mild light fluctuation after the addition of 1 min of bright light (800 µmol photons m^-2^ s^-1^) to every 5 min of low light (80 µmol photons m^-2^ s^-1^) and (**B**) severe light fluctuation after the addition of 1 min of stronger light (2,000 µmol photons m^-2^ s^-1^) to every 5 min of low light (20 µmol photons m^-2^ s^-1^).


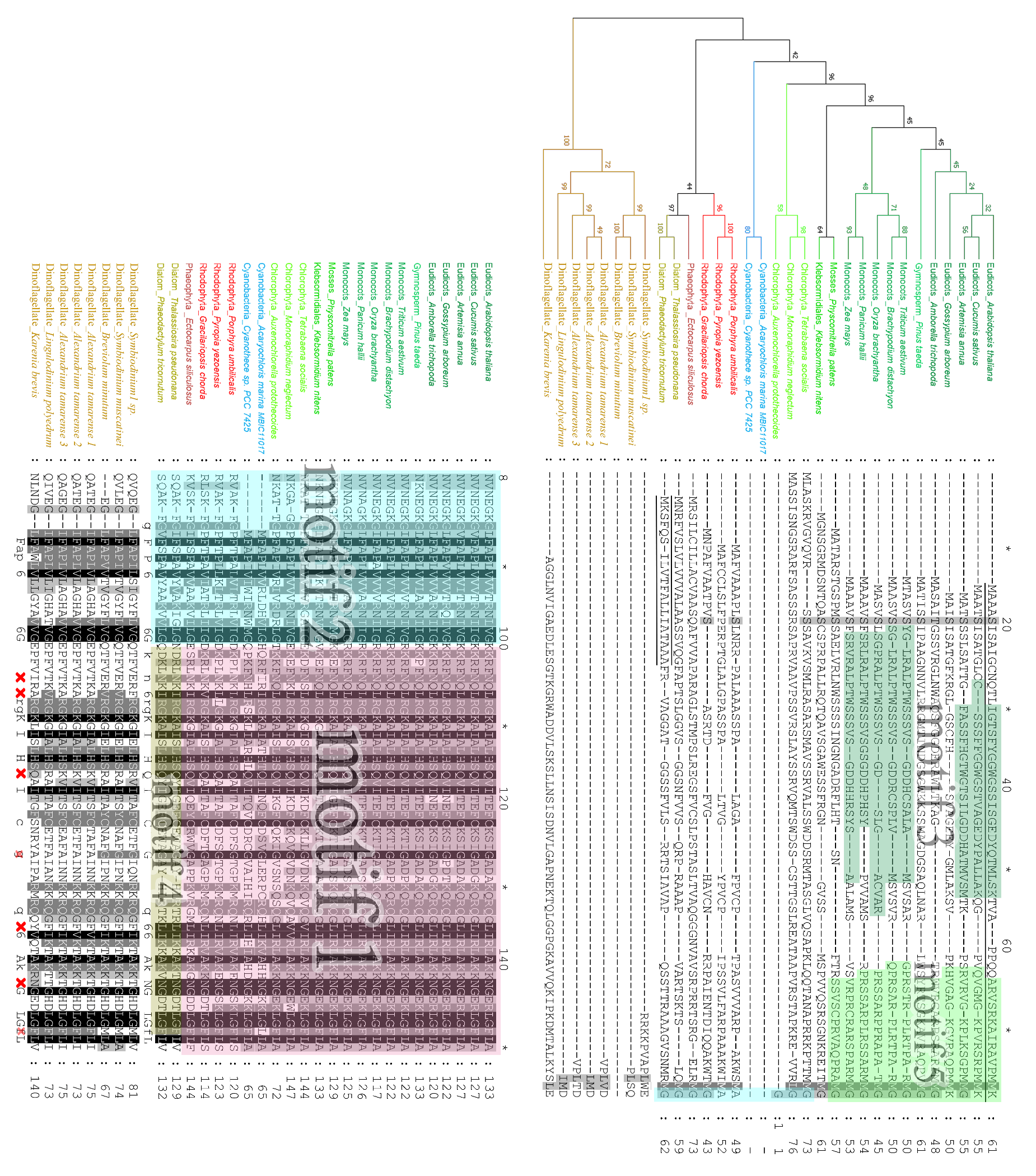


**Figure S2** Conserved sequences in, and evolutionary relationships of, PGR5 in plants and algae. Known signal peptide sequences are underlined. Motifs were identified using the MEME motif elicitation tool (Version 5.0.5). The missing conserved sites of dinoflagellates are shown with a red cross. The sequences are available in NCBI database (<http://www.ncbi.nlm.nih.gov>) or UniProtKB/TrEMBL database (<https://www.uniprot.org/>) as following. Eudicots: Arabidopsis thaliana (gi: 330250863), Cucumis sativus (gi: 164449273), Artemisia annua (gi: 1387830212), Gossypium arboreum (tr: I1ZIR9); Amborella: Amborella trichopoda (gi: 586688763); Gymnosperm: Pinus taeda (gi: 196168724); monocots: Triticum aestivum (gi: 393690734), Brachypodium distachyon (gi: 357144276), Oryza brachyantha (gi: 573956334), Panicum hallii (gi: 1435170242), Zea mays (gi: 1394909989); Mosses: Physcomitrella patens (gi: 1373914553); Klebsormidiales: Klebsormidium nitens (gi: 971519293); Chlorophyta: Tetrabaena socialis (gi: 1331346858), Monoraphidium neglectum (gi: 926775414), Auxenochlorella protothecoides (gi: 675355490); Cyanobacteria: Acaryochloris marina MBIC11017 (gi: 158308814), Cyanothece sp. PCC 7425 (gi: 219867356); Rhodophyta: Porphyra umbilicalis (gi: 1189386569), Pyropia yezoensis (tr: A1YSQ5), Gracilariopsis chorda (gi: 1395913517), Phaeophyta: Ectocarpus siliculosus (tr: D7G229); Diatom: Thalassiosira pseudonana (tr: B8C035), Phaeodactylum tricornutum (strain CCAP 1055/1) (tr: B7FVH9); Dinoflagellates: sequences were identified using NCBI-BLAST (BASF01, BGNK01, BGPT01, GAFO01, GBSC01, GFLM01, GFPM01, GHKS01, GICE01, IADN01, IADM01, VSDK0, PRJNA374496).


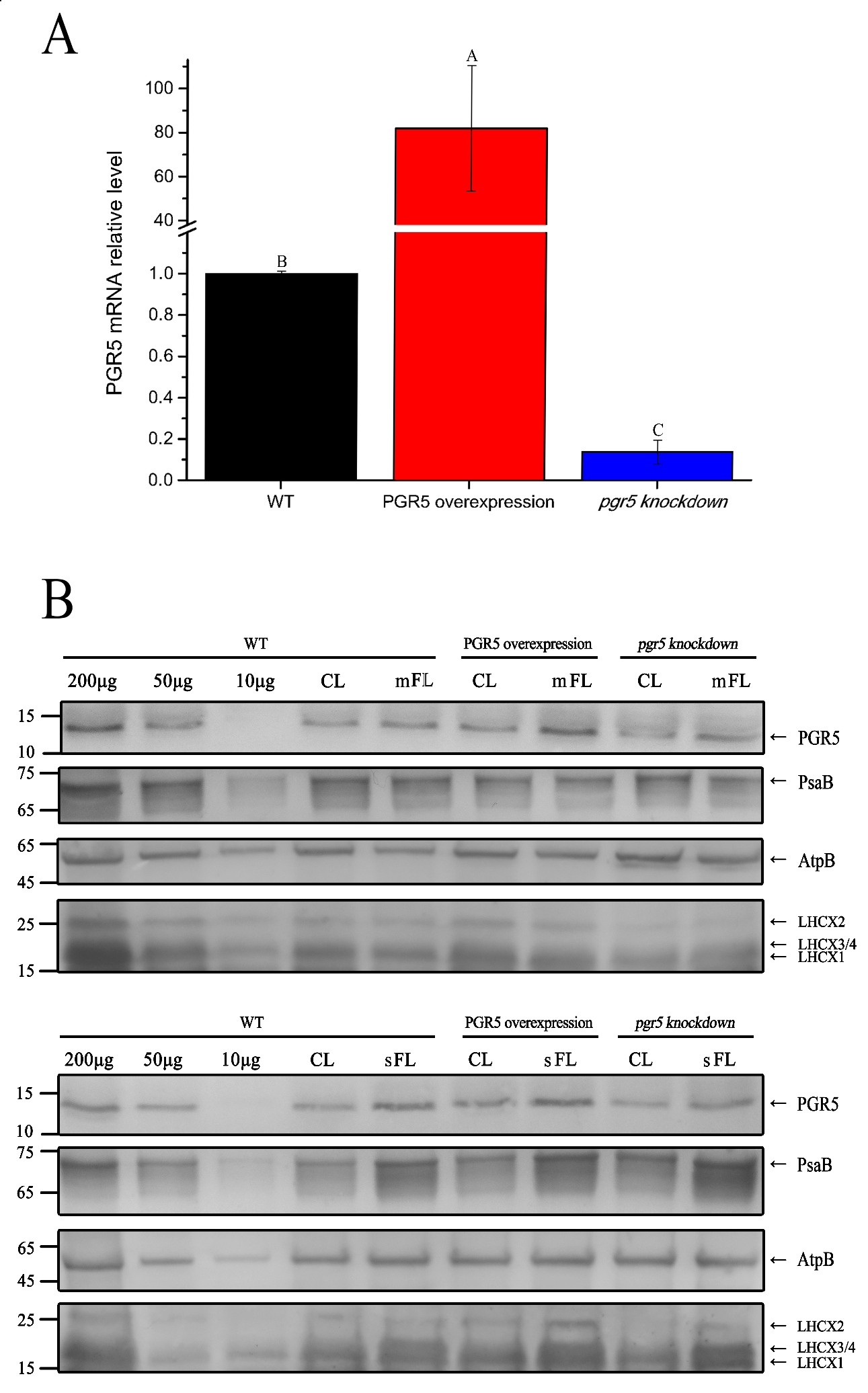


**Figure S3** Relative (**A**) RNA levels of PGR5 under 80 µmol photons m^-2^ s^-1^ and (**B**) thylakoid membrane protein levels (PGR5, PsaB, and AtpB) in the WT, 5OE-1, and 5KN-i1 under different light conditions. The protein was quantified using the BCA method (50 μg protein, ~3 μg Chl). CL: constant light under 80 µmol photons m^-2^ s^-1^; mFL: mildly fluctuating light: addition of 1 min of bright light (800 µmol photons m^-2^ s^-1^) to every 5 min of low light (80 µmol photons m^-2^ s^-1^); sFL: severely fluctuating light: addition of 1 min of stronger light (2,000 µmol photons m^-2^ s^-1^) to every 5 min of low light (20 µmol photons m^-2^ s^-1^). ANOVA was calculated by SPSS 23.0 (P<0.01).
